# Management Intensity and Topography Determined Plant Diversity in Vineyards

**DOI:** 10.1371/journal.pone.0076167

**Published:** 2013-10-01

**Authors:** Juri Nascimbene, Lorenzo Marini, Diego Ivan, Michela Zottini

**Affiliations:** 1 Department of Biology, University of Padova, Padova, Italy; 2 DAFNAE - Department of Agronomy, Food, Natural Resources, Animalsandthe Environment, University of Padova, Legnaro (Padova), Italy; University of Bern, Switzerland

## Abstract

Vineyards are amongst the most intensive forms of agriculture often resulting in simplified landscapes where semi-natural vegetation is restricted to small scattered patches. However, a tendency toward a more sustainable management is stimulating research on biodiversity in these poorly investigated agro-ecosystems. The main aim of this study was to test the effect on plant diversity of management intensity and topography in vineyards located in a homogenous intensive hilly landscape. Specifically, this study evaluated the role of slope, mowing and herbicide treatments frequency, and nitrogen supply in shaping plant diversity and composition of life-history traits. The study was carried out in 25 vineyards located in the area of the Conegliano-Valdobbiadene DOCG (Veneto, NE Italy). In each vineyard, 10 plots were placed and the abundance of all vascular plants was recorded in each plot. Linear multiple regression was used to test the effect of management and topography on plant diversity. Management intensity and topography were both relevant drivers of plant species diversity patterns in our vineyards. The two most important factors were slope and mowing frequency that respectively yielded positive and negative effects on plant diversity. A significant interaction between these two factors was also demonstrated, warning against the detrimental effects of increasing mowing intensity on steep slope where plant communities are more diverse. The response of plant communities to mowing frequency is mediated by a process of selection of resistant growth forms, such in the case of rosulate and reptant species. The other two management-related factors tested in this study, number of herbicide treatments and N fertilization, were less influential. In general, our study corroborates the idea that some simple changes in farming activities, which are compatible with grape production, should be encouraged for improving the natural and cultural value of the landscape by maintaining and improving wild plant diversity.

## Introduction

In the last decades, intensively cultivated areas have faced a severe loss of biodiversity [1]. However, the maintenance and improvement of biodiversity in agricultural landscapes is progressively more recognized as a key issue for improving human life-quality, promoting the cultural value of anthropogenic landscapes, and in general enhancing the provision of several ecosystem services [2].

Vineyards are amongst the most intensive forms of agriculture often resulting in simplified landscapes where semi-natural vegetation is restricted to small scattered patches. However, a recent trend among wine producers is to increasingly promote the cultural value of this landscape, recognizing the importance of coupling wine production with environmental quality. This tendency toward a more biodiversity friendly management is likely to reflect a general change in the mentality of vineyards owners (see e.g. 3) and is stimulating research on biodiversity in these poorly investigated agro-ecosystems [4,5]. The improvement of wild plant diversity in vineyards may sustain higher landscape biodiversity, providing refuge and food source for several vertebrates and arthropods, including those that are beneficial for pest control [3,6].

Management intensity is expected to influence plant diversity negatively although little research has been done in vineyards. On the other hand, some simple changes in farming activities, which are compatible with grape production, may potentially yield positive effects on the diversity of several taxonomic groups. It is therefore crucial to evaluate the role of those farming activities that are likely to influence plant diversity, such as the use of herbicides treatments, the frequency of mowing [7,8], mechanization and the supply of nitrogen fertilizers [9].

However, in hilly landscapes also topographic factors (e.g. slope, altitude, aspect) may influence plant diversity, even overriding the effect of management intensity and should therefore be taken into account [10]. Steep slopes tend to form shallow soils with lower nutrient and water availability [11]. If phosphorus and/or water limitation help to maintain a species-rich sward in chalk grassland, it would be expected that, at sites with varied topography, steeper slopes would be more resistant to change in vegetation composition than ﬂatter areas. However, it is still unknown the potential interaction between slope and other management practices such as mowing and herbicide treatments.

Hence, the main aim of this study was to test the effect on plant diversity of management intensity and topography in vineyards located in a homogenous intensive hilly landscape. Specifically, this study will evaluate the role of slope, mowing and herbicide treatments frequency, and nitrogen supply in shaping plant diversity and composition of life-history traits. We tested the effect of these factors on the abundance of rosulate and reptant species that are expected to be advantaged in more intensively managed sites. Specifically, these species are expected to be more resistant to mowing frequency [7,12,13] and this should be reflected by a higher relative cover in more frequently mowed vineyards. Moreover, this management-related pattern is expected to have a negative effect on community evenness since the dominance of rosulate and reptant species should hinder the establishment and development of less competitive plants.

## Methods

### Ethics statement

All necessary permits were obtained for the described field studies. In particular, we thank the owners of the vineyards who kindly allowed us to work in their fields and provided information on management practices.

### Study area

**Figure 1:**
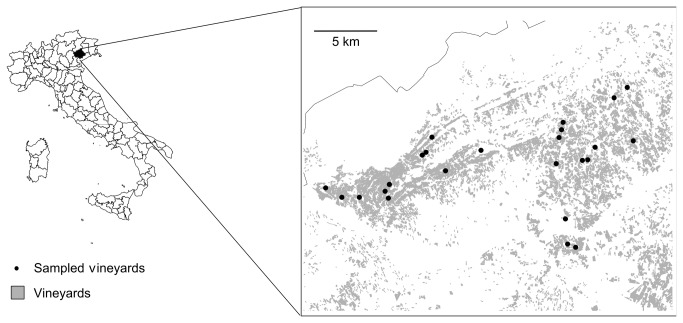
Study area and distribution of the 25 vineyards in the Treviso province (NE Italy).

The study was carried out in the area of the Conegliano-Valdobbiadene DOCG including 6100 ha of vineyards in the northern part of the province of Treviso (Veneto, NE Italy, N 45°52’40’’, E 12°17’5’’; Figure 1). This area is characterized by a hilly landscape where altitude ranges between 70 and 450 m, annual precipitation is between 900 and 1000 mm and mean annual temperature is 11°C. This hilly landscape is intensively cultivated with vineyards while semi-natural vegetation is restricted to small scattered forest patches or hedgerows. The cultivated area is composed by small vineyards, usually between 1 and 2 ha, belonging to several owners. This fragmented arrangement of the ownerships implies that management practices are not homogeneous at the landscape scale, strongly depending on the attitude of each single owner, and may vary even between adjacent vineyards. In particular, the control of weeds may have different intensity depending on mowing frequency and the use of herbicides. Also nitrogen supply is not constant, even if nitrogen input is generally low.

### Sampling design

Twenty-five vineyards belonging to different owners were selected to represent the whole geographical range of the Conegliano-Valdobbiadene DOCG and the gradient of management intensity (from intensive to extensive) and slope conditions (Table 1). The selection process was based on the database of the Conegliano-Valdobbiadene DOCG Consortium and information retrieved through farmers’ interviews. We interviewed c. 300 farmers to explore the management practices applied in the region. Of these only c. 60 agreed to share information with us and among them we selected the 25 vineyards to test four factors: mowing frequency, use of herbicide, nitrogen supply, and slope. These factors were selected based on the current knowledge on the main drivers of herbaceous plant diversity. Since we were interested in disentangling the effects of different management practices (mowing, use of herbicides, and nitrogen supply) and slope, vineyards were selected to keep as much as possible low collinearity between our predictors (all correlation <0.4). This allowed us to clearly separate the effect of each factor and test interactions. Mowing techniques included both manual devices such as string trimmer and mechanical devices mounted on tractors such as mowing bars and grass choppers. On average the vineyards manually mown present a significant (P<0.01) steeper slope (n=6, mean slope=75%, SE=5.22%) than the vineyard mown with the tractor (n=19, mean slope=22%, SE=4.78%). A potential effect of slope could therefore include also an additional effect of the mowing technique. The strong correlation between slope and mowing techniques did not allow selecting vineyards in order to keep the two factors statistically independent. Moreover, vineyards were selected to reduce difference in altitudes between the sites. Further criteria to include a vineyard in the study were an age above 10 years and a minimum area of 1 ha. All the vineyards have spontaneous vegetation and farmers did not sow any seed mix for at least 10 years.

**Table 1:** Average and range values of the main topographical and management related factors characterizing the 25 vineyards included in this study.

***Topographical factors***	**Mean±SD**	**Range (Min-Max)**
Altitude (m)	190±58	100-350
Slope (%)	34±30	0-90
Aspect (°)	185±74	76-340
***Management factors***		
N of mowing treatments yr^-1^	3.6±1	2-6
N of herbicide treatments yr^-1^	0.8±0,9	0-3
Total N (Kg ha^-1^)	18±25	0-70

Vascular plants were sampled once between April 2nd and 23th, 2012 before any management interventions. In each vineyard, 10 1 m x 1 m plots were randomly placed in the central part of the cultivated area in the field between grape rows. Within each plot, all vascular plants were recorded and for each species the abundance was visually estimated using 5% cover classes. Herbaceous species were further classified according to [14] as follows: perennial reptant and rosulate species, perennial species with erect stem, and summer annuals which overwinter by means of generative diasporas.

### Data analysis

As response variables we considered the additive components of diversity: alpha (α), beta (β) and gamma (γ) [15-17]. In this work, γ represents the total number of species found in vineyard, α represents the mean number of species at the plot level in each vineyard, and β was calculated as γ-α, giving estimates of the heterogeneity of the plant biota within each vineyard. β-diversity was expressed as a proportion of the total number of species (β/γ). We also computed community evenness using the E_var_ index [18]. This index has been chose due to its mathematical independence from species richness. We also tested the effect of our environmental predictors in selecting plant life forms indicative of management intensity (rosulate and reptant species).

To test the effect of management and topography on the above mentioned response variables we used linear multiple regression. The models initially included vineyard slope, mowing frequency, number of herbicide treatments and N fertilization. For the vineyard with slope steeper than 5% we tested in preliminary analyses the effect of aspect. As aspect was never associated with our response variables we omitted this predictor from the analyses presented here. All the predictors were centred to mean 0 and standard deviation 1. The slopes can therefore be used to evaluate the relative strength of the effect of the single variables. The models were simplified using a backward deletion procedure with P<0.05. The multiple regression was estimated using the lm() function in R, version 2.12.1 [19]. We could use P-values and traditional hypothesis testing on the slope due to the low collinearity between the predictors. A preliminary analysis using an information-theoretic approach based on AICc yielded very similar results and was therefore not presented here [20] (see electronic Text S1, Table S1).

## Results

A total of 141 species were found (see electronic Table S2). The mean number of species per vineyard (γ-diversity) was 35.2±12.7 (range: 19-69), the mean number of species per plot (α-diversity) was 13.4±3.3 (range: 8.8-21.3), and the mean heterogeneity (β-diversity) was 21.7±10 (range: 10.2-50). The mean value of evenness was 0.39±0.06, ranging between 0.27 and 0.55.

**Figure 2:**
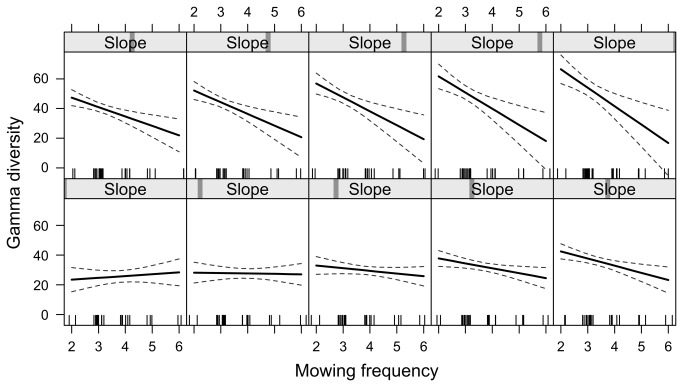
Interaction between vineyard slope and mowing frequency. The ﬁtted line is a general linear model estimate while the dashed lines indicated the intervals of conﬁdence (95%). The tick marks on the x-axis showed the values of the explanatory variables. Panels were drawn using the “effect” function from the library “effects” in R.

Both management intensity and topography had a significant effect on plant diversity (Table 2). In particular, α-diversity was negatively influenced by mowing frequency and fertilization while we found a marginal positive effect of slope. Vineyard species richness (γ-diversity) was negatively affected by mowing frequency and positively affected by slope. We also found a significant interaction between slope and mowing frequency, i.e. the negative effect of mowing was evident only on the steep slopes (Figure 2). Proportional β-diversity was also enhanced by steep slopes while no clear effect of management was found. Finally, evenness increased with increasing slope and declined with the number of herbicide treatments.

**Table 2:** Results of the multiple regression models testing the effect of vineyard slope, mowing frequency, herbicide treatment and fertilization on the four measures of diversity.

	**(b) Gamma**			**(a) Alpha**			**(c) Beta (%)**			**(d) E_var_**		
	**b**	**SE**	**P**	**b**	**SE**	**P**	**b**	**SE**	**P**	**b**	**SE**	**P**
Intercept	33.396	1.310	<0.01	13.46	0.44	<0.01	60.23	0.91	<0.01	0.39	0.01	<0.01
Slope	6.813	1.429	<0.01	0.99	0.49	0.05	4.24	0.93	<0.01	0.037	0.01	<0.01
Mowing	-4.319	1.501	<0.01	-1.96	0.49	<0.01	-	-	-	-	-	-
Herbicide	-	-	-	-	-	-	-	-	-	-0.024	0.01	0.02
Total N	-	-	-	-0.97	0.45	0.04	-	-	-	-	-	-
Slope x Mowing	-4.851	1.625	<0.01	-	-	-	-	-	-	-	-	-

The models were simplified with a backward deletion procedure (P<0.05). The interactions Slope x Herbicide and Slope x Total N were never retained in the models.

**Figure 3:**
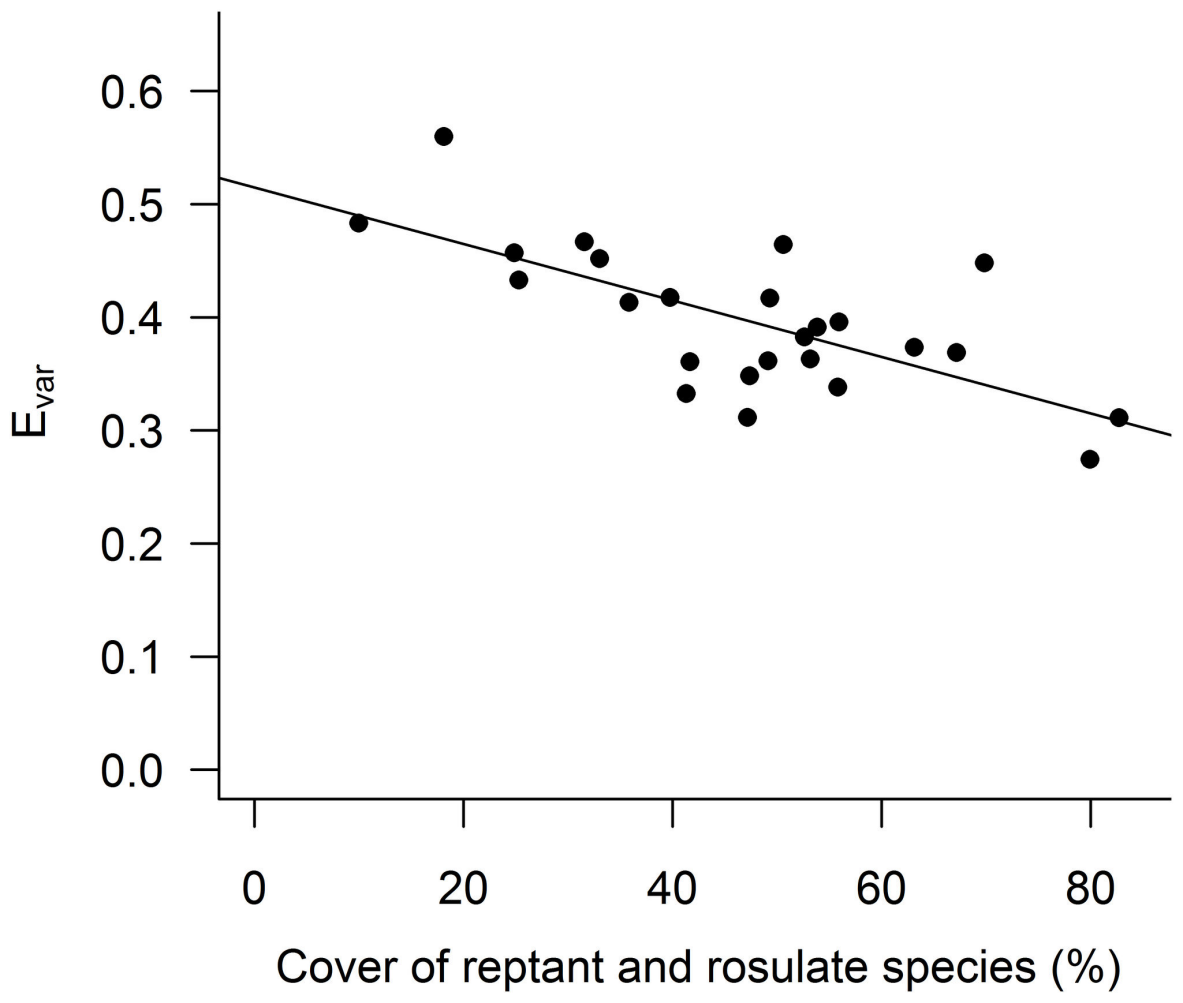
Relationship between the cover of rosulate and reptant species and species evenness. The line indicates a significant linear regression (P<0.01, R^2^=47.9).

The multiple regression analyses testing the effect of management intensity and slope on cover of rosulate and reptant species yielded significant results. In particular, the cover of rosulate and reptant species increased in more frequently mowed vineyards (b=8.34, SE=2.50, P<0.01), while their relative cover decreased with increasing slope (b=-8.80, SE=2.50, P<0.01). The dominance of rosulate and reptant species in more intensively managed and flat sites had a negative influence on the evenness of plant communities (Figure 3).

## Discussion

Management intensity and topography are both relevant drivers of plant species diversity patterns in our vineyards, confirming results already available for other types of agro-ecosystems [9].

The two most important factors are slope and mowing frequency that respectively yielded positive and negative effects on different measures of plant diversity. At our best knowledge, this is the first time that a significant interaction between these two factors in determining local plant diversity is also demonstrated, providing new insights for effective management practices to promote plant diversity. In particular, this result warns against the detrimental effects of increasing mowing intensity on steep slope (slope higher than 40%) where plant communities are more diverse. On the contrary, increasing mowing intensity may be not detrimental to plant diversity in flat sites where the species pool is poorer. These results predict that the maintenance of high plant diversity in our study area is mainly related to the management intensity applied to vineyards on steep slopes where low mowing frequency is recommended. As the steep slopes were more often mown with manual devices than flat areas, the lower impact of this mowing technique could have contributed to explain the interaction between slope and mowing frequency. Steeper slopes might be buffered to some extent against invasion by more competitive species, probably due to edaphic factors including low phosphorus availability [10].

The analysis of plant traits clarifies the mechanism that is behind the observed effect of mowing frequency on plant diversity. The response of plant communities to mowing frequency is mediated by a process of selection of resistant growth forms, such in the case of rosulate and reptant species [7,12,13,21]. These species tend to cover a large amount of the available surface hindering the establishment of plants that are less tolerant to mowing. This process is also reflected by the negative effect of the increasing cover of rosultae and reptant species on the evenness of plant communities, indicating the tendency of these resistant species to dominate in more disturbed sites. The decrease of community evenness is therefore a suitable indicator to evaluate the effects of mowing. However, results also suggest that in vineyards on steep slopes this mechanism is likely to be less efficient, preserving these vineyards from a severe homogenization of plant communities in terms of plant traits. This pattern is probably related to the fact that plants on steep slopes are subjected to more limiting factors other than management [10,22] that prevent the selection of a few growth forms and the dominance of few species.

The other two management-related factors tested in this study, number of herbicide treatments and N fertilization, are less influential on plant diversity in our vineyards. The influence of herbicide treatments is significant only for community evenness, suggesting a similar mechanism of plant selection as for mowing frequency, resulting in a simplified community dominated by resistant species. The absence of a significant effect of herbicide treatments on the additive components of diversity (alpha, beta and gamma) is likely to reflect the fact that in our study area herbicides are only applied to a restricted zone under the grape rows (c. 50-60 cm), while the fields between the rows, where our plots were placed, are not directly sprayed.

Nitrogen fertilization has a negative effect only on the mean number of species per plot (alpha-diversity), but does not affect the other components of diversity. In particular it does not cause negative effect on the total number of species at the vineyard level, supporting the idea that the need to decrease nitrogen supply is not a priority for effectively enhancing plant diversity in this context. Interestingly, in other agro-ecosystems, this factor is among the main management-related drivers of plant diversity [22]. In these cases, however, nitrogen inputs are higher by orders of magnitude compared with our vineyards where nitrogen supply is generally low and even not constant along time in a given site. The management of nitrogen fertilization is indeed highly variable even within a single vineyard and is not considered a priority, as in the case of mowing, by the owners.

In general, our study corroborates the idea that some simple changes in farming activities, which are compatible with grape production, should be encouraged for improving the natural and cultural value of the area of the Conegliano-Valdobbiadene DOCG, by maintaining and improving wild plant diversity. In particular, mowing frequency should be low (e.g. 2-3 times per year), especially on steep slopes where plant communities are more diverse. Despite its marginal effect on plant diversity, also the use of herbicides should be reduced, contributing to improve the aesthetic value of the landscape. These measures are likely to reduce management costs and to yield positive effects on the diversity of several taxonomic groups, including organisms that benefit ecosystem services such as pollination, biological control and grape resistance to pathogens.

## Supporting Information

Table S1 **Plausible candidate models (within 2 ∆AIC of top model) showing the effect of vineyard slope, mowing frequency, herbicide treatment and fertilization, separately for (a) gamma-diversity, (b) alpha, (c) beta (%), and evenness (Evar).** Models are ranked according to their second-order Akaike’s information criterion (AICc). Parameter estimates, and model weight (w_i_) are reported. All explanatory variables were standardized (mean=0, SD=1).
(DOCX)

Table S2 **Frequency of the species (i.e. number of plots in which the species occurred) recorded in the 25 vineyards.**
(DOCX)

Text S1 **Multi-model inference.** To test the consistency of our results we also used multi-model inference analyses whose method is explained in this supporting information.
(DOCX)
