# Supplementary material for: Management Intensity and Topography Determined Plant Diversity in Vineyards
Source: PLoS One. 2013 Oct 1;8(10):e76167. doi: 10.1371/journal.pone.0076167 (PMC3788025; doi:10.1371/journal.pone.0076167)
Supplement: Table S1 — Plausible candidate models (within 2 ∆AIC of top model) showing the effect of vineyard slope, mowing frequency, herbicide treatment and fertilization, separately for (a) gamma-diversity, (b) alpha, (c) beta (%), and evenness (Evar). Models are ranked according to their second-order Akaike’s information criterion (AICc). Parameter estimates, and model weight (wi) are reported. All explanatory variables were standardized (mean=0, SD=1). (DOCX) [file pone.0076167.s001.docx]

SUPPORTING INFORMATION

**Table S1** Plausible candidate models (within 2 ∆AIC of top model)showing the effect of vineyard slope, mowing frequency, herbicide treatment and fertilization, separately for (a) gamma-diversity, (b) alpha, (c) beta (%) and evenness (Evar). Models are ranked according to their second-order Akaike’s information criterion (AICc). Parameter estimates, and model weight (w_i_) are reported. All explanatory variables were standardized (mean=0, SD=1).

|  | **Intercept** | **Herbicide** | **Fertilization** | **Slope** | **Mowing frequency** | **Slope x**  **Mowing frequency** |  | **ΔAICc** | **Model weight** |
| --- | --- | --- | --- | --- | --- | --- | --- | --- | --- |
| **Gamma-diversity** |  |  |  |  |  |  |  |  |  |
|  | 33.396 | -3.830 |  | 6.813 | -4.319 | -4.851 |  | 0.000 | 0.444 |
|  | 33.396 | -3.495 | -2.020 | 6.599 | -4.780 | -4.850 |  | 0.190 | 0.404 |
| **Alpha-diversity** |  |  |  |  |  |  |  |  |  |
|  | 13.090 |  | -0.964 | 0.648 | -2.249 | -1.017 |  | 0.000 | 0.176 |
|  | 13.468 |  | -0.973 | 0.990 | -1.962 |  |  | 0.010 | 0.175 |
|  | 13.468 | -0.719 | -0.872 | 1.139 | -1.624 |  |  | 0.919 | 0.111 |
|  | 13.468 |  | -1.036 |  | -2.353 |  |  | 1.327 | 0.090 |
|  | 13.111 | -0.663 | -0.872 | 0.805 | -1.921 | -0.959 |  | 1.393 | 0.088 |
|  | 13.468 | -0.863 |  | 1.232 | -1.425 |  |  | 1.781 | 0.072 |
|  | 13.468 |  |  | 1.061 | -1.812 |  |  | 1.812 | 0.071 |
| **Beta-diversity** |  |  |  |  |  |  |  |  |  |
|  | 60.232 |  |  | 4.242 |  |  |  | 0.000 | 0.402 |
|  | 60.232 | -0.896 |  | 4.263 |  |  |  | 1.829 | 0.161 |
| **Evenness** |  |  |  |  |  |  |  |  |  |
|  | 0.397 | -0.025 |  | 0.037 |  |  |  | 0.000 | 0.249 |
|  | 0.397 | -0.019 |  | 0.031 | -0.016 |  |  | 1.132 | 0.141 |
|  | 0.397 |  |  | 0.027 | -0.024 |  |  | 1.225 | 0.135 |
|  | 0.389 |  |  | 0.020 | -0.030 | -0.021 |  | 1.648 | 0.109 |
|  | 0.390 | -0.017 |  | 0.024 | -0.022 | -0.020 |  | 1.986 | 0.092 |
