# Supplementary material for: Management Intensity and Topography Determined Plant Diversity in Vineyards
Source: PLoS One. 2013 Oct 1;8(10):e76167. doi: 10.1371/journal.pone.0076167 (PMC3788025; doi:10.1371/journal.pone.0076167)
Supplement: Table S2 — Frequency of the species (i.e. number of plots in which the species occurred) recorded in the 25 vineyards. (DOCX) [file pone.0076167.s002.docx]

SUPPORTING INFORMATION

**Table S2** Frequency of the species (i.e. number of plots in which the species occurred) recorded in the 25 vineyards.

| **Species name** | **Frequency** |
| --- | --- |
| Achillea millefolium L. s.l. | 13 |
| Aegopodium podagraria L. | 15 |
| Ajuga reptans L. | 27 |
| Allium vineale L. | 17 |
| Anemone nemorosa L. | 12 |
| Anemone trifolia L. s.l. | 1 |
| Anthoxanthum odoratum L. s.l. | 14 |
| Aristolochia pallida Willd. | 12 |
| Arrhenatherum elatius (L.) P. Beauv. ex J.& C. Presl s.l. | 12 |
| Artemisia vulgaris L. | 6 |
| Arum italicum Mill. subsp. italicum | 1 |
| Asparagus tenuifolius Lam. | 1 |
| Astragalus glycyphyllos L. | 2 |
| Bellis perennis L. | 102 |
| Brachypodium pinnatum (L.) P. Beauv. | 8 |
| Bromus hordeaceus L. s.l. | 3 |
| Bromus sterilis L. | 36 |
| Calepina irregularis (Asso) Thell. | 4 |
| Calystegia sepium (L.) R. Br. subsp. sepium | 83 |
| Capsella bursa-pastoris (L.) Medik. subsp. bursa-pastoris | 32 |
| Cardamine hirsuta L. | 64 |
| Carex canescens L. | 12 |
| Carex caryophyllea Latourr. | 2 |
| Carex flacca Schreb. s.l. | 1 |
| Carex spicata Huds. | 1 |
| Centaurea nigrescens Willd. s.l. | 12 |
| Cerastium holosteoides Fr. | 66 |
| Chelidonium majus L. | 1 |
| Chenopodium album L. s.l. | 1 |
| Cirsium arvense (L.) Scop. | 2 |
| Clinopodium vulgare L. s.l. | 1 |
| Colchicum autumnale L. | 7 |
| Convolvulus arvensis L. | 5 |
| Cornus sanguinea L. s.l. | 1 |
| Crepis biennis L. | 27 |
| Cruciata glabra (L.) Ehrend. s.l. | 7 |
| Cynodon dactylon (L.) Pers. | 59 |
| Dactylis glomerata L. s.l. | 14 |
| Daucus carota L. s.l. | 9 |
| Elymus repens (L.) Gould. subsp. repens | 85 |
| Equisetum arvense L. | 5 |
| Erigeron annuus (L.) Desf. | 28 |
| Erodium cicutarium (L.) L'Hér. | 1 |
| Euphorbia cyparissias L. | 6 |
| Euphorbia helioscopia L. subsp. helioscopia | 42 |
| Festuca gr. rupicola | 1 |
| Festuca sp. | 2 |
| Filipendula vulgaris Moench | 3 |
| Fragaria vesca L. subsp. vesca | 6 |
| Fumaria officinalis L. s.l. | 3 |
| Galium mollugo L. s.l. | 56 |
| Galium verum L. s.l. | 1 |
| Geranium dissectum L. | 44 |
| Geranium molle L. | 68 |
| Geranium rotundifolium L. | 4 |
| Gladiolus italicus Mill. | 1 |
| Glechoma hederacea L. | 20 |
| Hedera helix L. s.l. | 2 |
| Helianthemum nummularium (L.) Mill. s.l. | 1 |
| Helleborus viridis L. subsp. Viridis | 11 |
| Heracleum sphondylium L. s.l. | 4 |
| Hieracium pilosella L. | 1 |
| Holcus lanatus L. | 15 |
| Hordeum murinum L. s.l. | 10 |
| Hypochoeris radicata L. | 7 |
| Knautia drymeia Heuff. s.l. | 5 |
| Lactuca saligna L. | 7 |
| Lamium album L. subsp. album | 8 |
| Lamium amplexicaule L. | 1 |
| Lamium orvala L. | 5 |
| Lamium purpureum L. | 72 |
| Lathyrus pratensis L. s.l. | 8 |
| Leontodon hispidus L. | 3 |
| Leucanthemum vulgare Lam. subsp. vulgare | 1 |
| Lolium multiflorum Lam. s.l. | 7 |
| Lotus corniculatus L. s.l. | 15 |
| Lysimachia nummularia L. | 7 |
| Malva sylvestris L. subsp. sylvestris | 4 |
| Medicago lupulina L. | 2 |
| Medicago sativa L. | 5 |
| Mentha arvensis L. | 4 |
| Mentha suaveolens Ehrh. s.l. | 1 |
| Mercurialis annua L. | 1 |
| Muscari neglectum Guss. ex Ten. | 10 |
| Myosotis arvensis (L.) Hill subsp. arvensis | 1 |
| Ophioglossum vulgatum L. | 2 |
| Ornithogalum umbellatum L. | 3 |
| Oxalis corniculata L. | 19 |
| Papaver rhoeas L subsp. rhoeas | 1 |
| Parietaria officinalis L. | 3 |
| Peucedanum oreoselinum (L.) Moench. | 1 |
| Pimpinella major (L.) Huds. | 6 |
| Plantago lanceolata L. | 107 |
| Plantago major L. s.l. | 78 |
| Poa annua L. | 24 |
| Poa compressa L. | 154 |
| Poa pratensis L. | 25 |
| Poa trivialis L. | 181 |
| Polygonum aviculare L. s.l. | 10 |
| Potentilla reptans L. | 125 |
| Primula vulgaris Huds. subsp. vulgaris | 3 |
| Prunus avium L. subsp. avium | 1 |
| Pulmonaria officinalis L. | 5 |
| Ranunculus acris L. s.l. | 70 |
| Ranunculus arvensis L. | 1 |
| Ranunculus bulbosus L. | 20 |
| Ranunculus ficaria L. s.l. | 1 |
| Ranunculus repens L. | 79 |
| Rumex acetosa L. subsp. acetosa | 41 |
| Rumex obtusifolius L. s.l. | 80 |
| Salvia pratensis L. s.l. | 9 |
| Sanguisorba minor Scop. s.l. | 1 |
| Senecio vulgaris L. | 10 |
| Sherardia arvensis L. | 3 |
| Silene flos-cuculi (L.) Clairv. | 7 |
| Silene latifolia Poir. subsp. Alba (Mill.) Greuter & Burdet | 45 |
| Silene nutans L. s.l. | 1 |
| Silene vulgaris (Moench) Garcke s.l. | 3 |
| Solanum nigrum L. | 3 |
| Sonchus oleraceus L. | 25 |
| Stachys officinalis (L.) Trevis. | 1 |
| Stellaria media (L.) Vill. s.l. | 113 |
| Symphytum officinale L. s.l. | 8 |
| Symphytum tuberosum L. subsp. angustifolium (A. Kern.) Nyman | 19 |
| Tamus communis L. | 2 |
| Taraxacum officinale Weber (group) | 200 |
| Teucrium chamaedrys L. s.l. | 1 |
| Thymus sp. | 2 |
| Trifolium pratense L. s.l. | 79 |
| Trifolium repens L. s.l. | 121 |
| Urtica dioica L. subsp. dioica | 60 |
| Valerianella locusta (L.) Laterr. | 50 |
| Verbena officinalis L. | 4 |
| Veronica arvensis L. | 79 |
| Veronica chamaedrys L. s.l. | 22 |
| Veronica hederifolia L. s.l. | 11 |
| Veronica persica Poir. | 130 |
| Vicia hirsuta (L.) Gray | 3 |
| Vicia sativa L. s.l. | 12 |
| Vinca minor L. | 7 |
| Viola suavis M. Bieb. subsp. suavis | 9 |
