# Supplementary material for: Management Intensity and Topography Determined Plant Diversity in Vineyards
Source: PLoS One. 2013 Oct 1;8(10):e76167. doi: 10.1371/journal.pone.0076167 (PMC3788025; doi:10.1371/journal.pone.0076167)
Supplement: Text S1 — Multi-model inference. To test the consistency of our results we also used multi-model inference analyses whose method is explained in this supporting information. (DOCX) [file pone.0076167.s003.docx]

SUPPORTING INFORMATION

**Text S1**

**Multi-model inference**

Due to the relatively low number of replicates (n=25) and the relatively high number of potential predictors, we also used multi-model inference within an information-theoretic framework to evaluate the role of the selected variables in explaining patterns of plant diversity (Burnham and Anderson 2002). We used ordinary least square multiple linear regression to estimate model parameters as model residuals approximated a normal distribution. As response variables we considered the additive components of diversity: alpha (á), beta (â) and gamma (ã) (Allan 1975; Lande 1996; Roschewitz et al. 2005) and community evenness using the E_var_ index (Smith & Wilson 1996). Our information-theoretic approach compared the fit of all the possible candidate models obtained by the combination of the four predictors using second-order Akaike’s information criterion (AICc). The AICc is a measure of relative model fit, proportional to the likelihood of the model and the number of parameters used to generate it. The best fitting model is the one with the lowest AICc. In a set of n models each model i can be ranked using its difference in AICc score with the best-fitting model (ΔAICc_i_ = AICc_i_–AICc minimum). The difference in AICc values indicates the relative support for the different models. A model is usually considered plausible if its ΔAICc is below 2 (Burnham and Anderson 2002). For each model i we also calculated an Akaike’s weight (w_i_), which is the probability that model i would be selected as the best fitting model if the data were collected again under identical circumstances (Burnham and Anderson 2002). Akaike’s weight should be interpreted as a measure of model selection uncertainty. The multi-model inference analyses were performed using the ‘MuMIn’ package (Barton 2010) implemented in R version 2.8.0 (R Development Core Team 2008).
